# Five-Year Changes in Pachydrusen with Late-Phase Hyperfluorescence on Indocyanine Green Angiography

**DOI:** 10.3390/jcm15082836

**Published:** 2026-04-09

**Authors:** Hiroyuki Kamao, Katsutoshi Goto, Kenichi Mizukawa, Ryutaro Hiraki, Atsushi Miki, Shuhei Kimura

**Affiliations:** 1 Department of Ophthalmology, Kawasaki Medical School, 577 Matsushima, Kurashiki 701-0114, Japan; k_goto@med.kawasaki-m.ac.jp (K.G.); hiraki@med.kawasaki-m.ac.jp (R.H.); kimuras@med.kawasaki-m.ac.jp (S.K.); 2 Shirai Eye Hospital, 1339 Takasecho, Kamitakase, Mitoyo 767-0001, Japan; mizu-p@shirai-hosp.or.jp

**Keywords:** pachydrusen, hyperfluorescence, indocyanine green angiography, neovascular age-related macular degeneration

## Abstract

**Background/Objectives:** Pachydrusen are a drusen subtype associated with the pachychoroid disease spectrum; however, their long-term natural history and pathophysiological significance remain unclear. We investigated 5-year morphological and topographic changes in pachydrusen using diagnostic criteria incorporating late-phase indocyanine green angiography (ICGA) hyperfluorescence. **Methods:** This retrospective observational study included fellow eyes with pachydrusen from patients with unilateral neovascular age-related macular degeneration. Pachydrusen were defined as sub-retinal pigment epithelium (RPE) deposits ≥ 125 µm in size with corresponding hyperfluorescence on late-phase ICGA. Lesion number, size, and spatial distribution (ETDRS grid and quadrant-based classification) were evaluated at baseline and 5 years. The incidence of macular neovascularization (MNV) and its colocalization with pachydrusen were assessed. **Results:** Among 57 fellow eyes with pachydrusen, incident MNV developed in 8 eyes (14.0%) during follow-up; the mean time to onset was 25.6 ± 16.3 months. No clear colocalization between pachydrusen and incident MNV was observed. Nineteen eyes completed the 5-year follow-up period. Pachydrusen were predominantly located outside the 6000 µm ETDRS grid at baseline (63.4%) and 5 years (66.3%), significantly exceeding the expected proportion based on the area ratio (*p* < 0.001). The lesions were most frequently observed in the superotemporal quadrant (52.6%). Over 5 years, 19.8% of the lesions increased in size, 67.2% remained stable, and 12.9% regressed; none of the regressed lesions were accompanied by RPE atrophy. **Conclusions:** Pachydrusen, defined as late-phase ICGA hyperfluorescence, was predominantly distributed outside the ETDRS grid with a superotemporal predilection and could increase or decrease over a 5-year follow-up period. No colocalization with MNV was observed, and no accompanying RPE atrophy after pachydrusen regression was identified, suggesting that late-phase ICGA–hyperfluorescent pachydrusen may represent a pathophysiology distinct from that of soft drusen.

## 1. Introduction

Drusen are extracellular deposits formed between the retinal pigment epithelium (RPE) and Bruch’s membrane [1]. Drusen are a hallmark of age-related macular degeneration (AMD), and soft drusen are known to enlarge, become confluent, and regress over time. Regression is frequently followed by RPE atrophy and progression to geographic atrophy (GA) [2].

Recently, pachydrusen have attracted attention as drusenoid deposits that differ from soft drusen regarding their morphology, distribution, and underlying pathophysiology [3]. Pachydrusen are considered a drusen subtype associated with pachychoroid disease [4] and are typically ≥ 125 µm in size, with well-defined yet irregular contours. They usually appear as yellow-white deposits scattered throughout the posterior pole. In late-phase indocyanine green angiography (ICGA), soft drusen show hypofluorescence [5], whereas pachydrusen frequently demonstrate hyperfluorescence [6]. In a population-based 5-year cohort study, no eyes developed RPE atrophy after pachydrusen regression, and no incident macular neovascularization (MNV) was observed [7]. Moreover, in the fellow eyes of patients with unilateral neovascular AMD (nAMD), the colocalization rate of MNV with drusen was 100% and 29% in eyes with soft drusen and pachydrusen, respectively, suggesting differences in the natural history and underlying mechanisms between these drusen subtypes [8]. Accordingly, international consensus statements have emphasized the importance of distinguishing pachydrusen from soft drusen [9]; however, the lack of standardized diagnostic criteria for pachydrusen limits accurate differentiation.

Most reports describing longitudinal morphological changes in pachydrusen rely primarily on color fundus photographs, which can be influenced by imaging conditions and background pigmentation [8,9,10,11]. In contrast, longitudinal studies that incorporate late-phase ICGA hyperfluorescence, which more clearly differentiates pachydrusen from soft drusen, into the diagnostic criteria remain limited. In this study, we evaluated the 5-year changes in the morphology and spatial distribution of pachydrusen in the fellow eyes (study eyes) of patients with unilateral nAMD using diagnostic criteria based on late-phase ICGA hyperfluorescence and morphological features. Furthermore, we assessed the 5-year incidence of MNV, their co-localization with pachydrusen, and the presence of RPE atrophy after pachydrusen regression.

## 2. Materials and Methods

### 2.1. Study Design

This was a single-center retrospective study performed at Kawasaki Medical School. We reviewed the medical records of consecutive treatment-naïve patients with unilateral nAMD who underwent treatment between January 2014 and December 2019. Information on age, sex, hypertension, diabetes mellitus, and smoking status was extracted from medical records. Smoking status was categorized as never-smoker or ever-smoker based on our published definition [12]. The exclusion criteria were as follows: prior laser photocoagulation or vitrectomy; high myopia (>−6 diopters), uveitis, or angioid streaks; and other ocular diseases that could affect outcomes in the study eye, including branch retinal vein occlusion, diabetic retinopathy, or glaucoma. Eyes with missing baseline or follow-up clinical or imaging data were excluded from the analysis.

### 2.2. Data Collection

**Figure 1:**
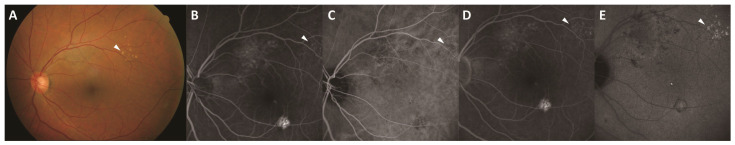
Representative case of typical pachydrusen in a 70-year-old man. All images (**A**–**E**) were obtained at baseline. (**A**) Color fundus photograph showing pachydrusen (white arrowhead). (**B**) Early-phase FA showing a hyperfluorescent area (white arrowhead) corresponding to pachydrusen. Typically, larger lesions indicate hyperfluorescence (window defect), whereas smaller lesions appear isofluorescent. (**C**) Early-phase ICGA showing a hypofluorescent area (white arrowhead) corresponding to pachydrusen. (**D**) Late-phase FA indicating a hyperfluorescent area without progressive staining or leakage (white arrowhead) corresponding to pachydrusen. (**E**) Late-phase ICGA showing hyperfluorescence corresponding to pachydrusen (white arrowhead). FA, fluorescein angiography; ICGA, indocyanine green angiography.

**Figure 2:**
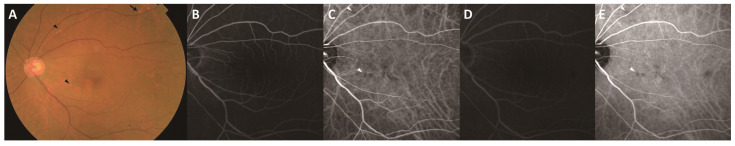
Representative excluded case in a 71-year-old man. All images (**A**–**E**) were obtained at baseline. (**A**) Color fundus photograph showing isolated yellow lesions ≥125 µm in size with well-defined borders, located outside the macula (black arrowheads and black arrow). The lesion indicated by the black arrow was excluded because it was not captured within the ICGA field of view and therefore could not be assessed. (**B**) Early-phase FA shows no obvious abnormalities. (**C**) Early-phase ICGA indicates hypofluorescent areas (white arrowheads) corresponding to the drusen. (**D**) Late-phase FA shows no obvious abnormalities. (**E**) Late-phase ICGA shows hypofluorescent areas (white arrowheads) corresponding to the drusen. These lesions were excluded because they showed no hyperfluorescence on late-phase ICGA. FA, fluorescein angiography; ICGA, indocyanine green angiography.

All patients received anti-vascular endothelial growth factor therapy using a treat-and-extend regimen. The fellow eye (study eye) underwent comprehensive ophthalmological examinations at regular intervals, including best-corrected visual acuity measurement, indirect ophthalmoscopy, slit-lamp biomicroscopy with a noncontact lens, color fundus photography, swept-source optical coherence tomography (OCT) (DRI OCT-1 Atlantis; Topcon Corporation, Tokyo, Japan), and fluorescein angiography (FA) and ICGA (HRA-2; Heidelberg Engineering GmbH, Dossenheim, Germany). Subfoveal choroidal thickness (SFCT) was measured using swept-source OCT, according to previously published methods [13]. Pachydrusen were assessed in all fellow eyes based on criteria described in a previous study [14]. Pachydrusen were defined as yellow-white deposits on color fundus photographs corresponding to sub-RPE deposits on OCT. Lesions were required to be ≥125 µm in size, with well-defined but irregular contours, and to be isolated or scattered across the posterior pole. On OCT B-scans, pachydrusen appeared as drusenoid deposits overlying the pachyvessels. Lesions were also required to show hyperfluorescence on late-phase ICGA. Pachydrusen were identified primarily on color fundus photographs, and lesions without corresponding late-phase ICGA hyperfluorescence were excluded from the analysis. Two blinded retinal specialists (H.K. and K.G.) independently assessed the presence or absence of pachydrusen, pachydrusen size, and colocalization between pachydrusen and incident MNV using color fundus photography, FA, and ICGA. These assessments were qualitative. Discrepancies were adjudicated by a senior grader (K.M.). Among lesions that were ultimately diagnosed as pachydrusen, the crude interobserver agreement rates for the diagnosis of pachydrusen were 96.8% at baseline and 96.0% at 5 years. Cohen’s kappa coefficients were low at both time points (baseline: κ = −0.015; 5 years: κ = −0.020), likely reflecting the highly imbalanced distribution of ratings, with very few lesions classified as pachydrusen-negative. Representative images of the included and excluded cases are shown in Figure 1 and Figure 2, respectively.

### 2.3. Outcome Measures

**Figure 3:**
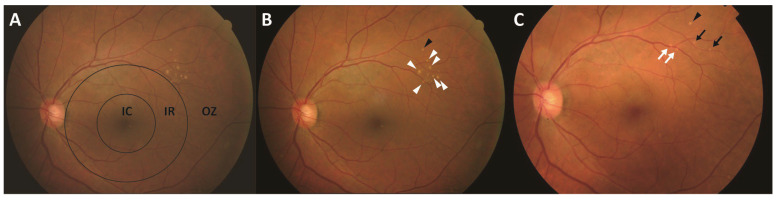
Representative case of typical pachydrusen in a 70-year-old man. Images (**A**,**B**) were obtained at baseline, and image (**C**) was obtained 5 years after baseline. (**A**) Color fundus photograph showing the concentric ring-based classification centered on the fovea. Two concentric circles with diameters of 3000 µm and 6000 µm subdivide the fundus into the following three regions: the inner circle, intermediate ring, and outer zone. The outer zone was graded to the visible extent of each image. In this case, all lesions were located in the outer zone. (**B**) At baseline, a black arrowhead indicates pachydrusen ≥ C1 in size that remained stable over 5 years, whereas white arrowheads show pachydrusen ≥ C1 that subsequently disappeared. (**C**) At 5 years, a black arrowhead indicates a pachydrusen lesion that remained stable, black arrows indicate lesions that increased in size (C0 to C1), and white arrows represent newly developed lesions. In this case, one lesion remained stable, four lesions increased in size, and six lesions regressed without associated RPE atrophy. IC, inner circle; IR, intermediate ring; OZ, outer zone.

Drusen location was evaluated using two classification schemes: concentric rings centered on the fovea and a quadrant-based classification. For the concentric ring-based classification (Figure 3), the fundus was subcategorized into three regions using two concentric circles centered on the fovea with diameters of 3000 µm and 6000 µm, respectively, similar to the Wisconsin age-related maculopathy grading system [15]: the inner circle, intermediate ring, and outer zone. The inner circle was defined as within the 3000 µm circle, the intermediate ring as between the 3000 µm and 6000 µm circles, and the outer zone as beyond the 6000 µm circle, graded to the visible extent of each image. For the quadrant-based classification, the fundus was classified into the following four quadrants centered on the fovea: superonasal, inferonasal, superotemporal, and inferotemporal. Lesion size was graded on color fundus photographs using standardized circles: C0 (63 µm), C1 (125 µm), C2 (175 µm), and C3 (250 µm) [15]. Longitudinal size changes were classified as increased, stable, or decreased based on changes in size category. For the baseline analysis, pachydrusen were classified as located inside or outside the 6000 µm diameter ETDRS grid. The pixel area of each region (inside vs. outside) was quantified on 30-degree HRA-2 images using the ImageJ software (version 1.54g; National Institutes of Health, Bethesda, MD, USA).

### 2.4. Statistical Analysis

Statistical analyses were performed using the JMP Pro 17 software (SAS Institute, Cary, NC, USA). Time-to-event analysis of incident MNV was performed using univariable Cox proportional hazards regression models, and hazard ratios (HRs) with 95% confidence intervals (CIs) were calculated. The proportion of pachydrusen located outside the grid was tested using a two-sided exact binomial test. The null probability (p_0_) was defined as the mean area ratio of the region outside the 6000 µm ETDRS grid to the total 30-degree HRA-2 image area (in pixels^2^). Continuous variables are presented as mean ± standard deviation, while categorical variables are expressed as counts and percentages. Baseline characteristics were compared between the stable and changed groups (increase in size or decrease in size) using Fisher’s exact test (two-sided) for categorical variables and the Wilcoxon rank-sum test for continuous variables. To evaluate the distribution of pachydrusen over time (i.e., the proportion located outside the ETDRS grid), a linear mixed-effects model was used to assess the time effect (baseline vs. 5 years) and inter-individual variability among patients (random effects). The distribution of pachydrusen among the four quadrants (superotemporal, inferotemporal, superonasal, and inferonasal) was analyzed using Pearson’s chi-square test. Additionally, the distribution of pachydrusen showing size changes (increase in size or decrease in size) across the four quadrants was evaluated using a linear mixed-effects model to account for within-patient clustering. Statistical significance was set at *p* < 0.05.

## 3. Results

### 3.1. Clinical Characteristics of the Study Population

In this study, pachydrusen were defined as drusen showing hyperfluorescence on late-phase ICGA. Drusen without late-phase ICGA hyperfluorescence, including those with pachydrusen-like morphology, were not classified as pachydrusen. Based on this definition, 94 eyes were categorized into the non-pachydrusen group. Overall, 57 treatment-naïve patients with unilateral nAMD, whose fellow eyes exhibited pachydrusen (*n* = 57), were included. The mean age was 73.9 ± 9.0 years; 37 and 20 patients were men and women, respectively. The mean follow-up duration for assessing the development of MNV in the overall cohort was 30.7 ± 24.3 months. During follow-up, MNV developed in eight eyes (14.0%). No colocalization between pachydrusen and incident MNV was observed in any of the eight eyes. The mean time to MNV onset was 25.6 ± 16.3 months (median, 25.4 months). The baseline demographic and clinical characteristics stratified based on the development of MNV are summarized in Table 1. Given that the follow-up duration varied among patients, and grouping was based on event occurrence, Table 1 presented descriptive statistics only, and no between-group comparisons were performed.

**Figure 4:**
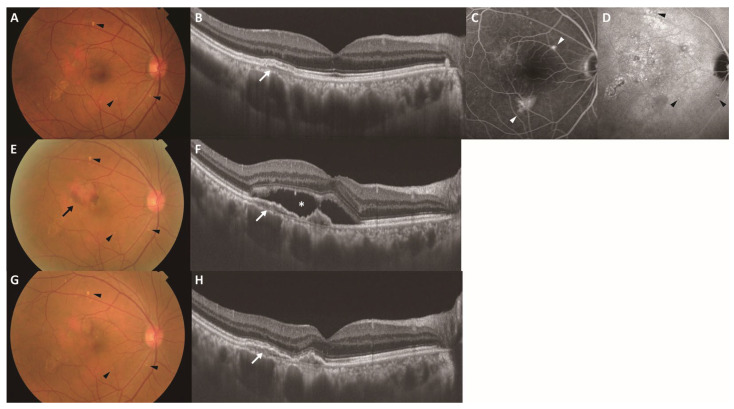
Representative case of incident MNV in the fellow eye (study eye) during follow-up in a 58-year-old man. Images (**A**–**D**) were obtained at baseline, images (**E**,**F**) were obtained 37 months after baseline (time of MNV onset), and images (**G**,**H**) were obtained 3 months after initiating intravitreal aflibercept. (**A**) Color fundus photograph showing pachydrusen (black arrowheads). (**B**) Swept-source OCT B-scan of the macula indicating a pigment epithelial detachment (white arrow) overlying a pachyvessel. (**C**) Late-phase FA depicting a hyperfluorescent area (white arrowheads) consistent with window defects, with no obvious FA abnormalities corresponding to the pachydrusen. (**D**) Late-phase ICGA showing hyperfluorescence corresponding to pachydrusen (black arrowheads). (**E**) Color fundus photograph indicating a yellow-white lesion surrounded by hemorrhage (black arrow), while the pachydrusen remained stable (black arrowheads). (**F**) Swept-source OCT B-scan of the macula showing a flat irregular pigment epithelial detachment (white arrow) with subretinal fluid (asterisk). (**G**) Color fundus photograph indicating the resolution of the hemorrhage, while the pachydrusen remained stable (black arrowheads). (**H**) Swept-source OCT B-scan of the macula showing a flat irregular pigment epithelial detachment (white arrow) without subretinal fluid. FA, fluorescein angiography; ICGA, indocyanine green angiography; MNV, macular neovascularization; OCT, optical coherence tomography.

**Figure 5:**
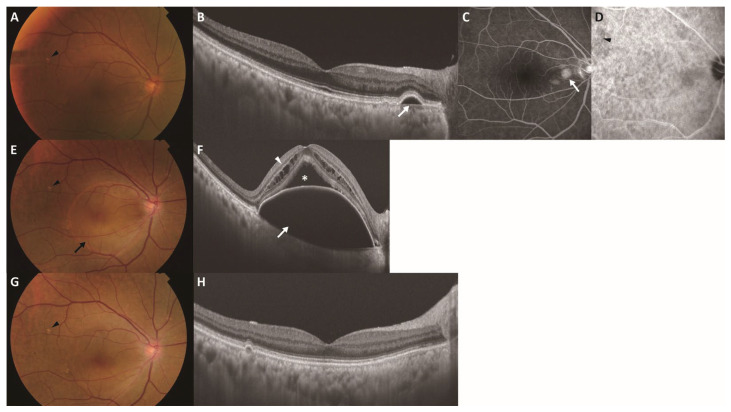
Representative case of incident MNV in the fellow eye (study eye) during follow-up in a 72-year-old woman. Images (**A**–**D**) were obtained at baseline, images (**E**,**F**) were obtained 31 months after baseline (time of MNV onset), and images (**G**,**H**) were obtained 1 month after initiating intravitreal brolucizumab. (**A**) Color fundus photograph representing pachydrusen (black arrowhead). (**B**) Swept-source OCT B-scan of the macula indicating a serous pigment epithelial detachment (white arrow) adjacent to the optic disk. (**C**) Late-phase FA showing a hyperfluorescent area (white arrow) consistent with pooling, with no abnormal FA findings corresponding to the pachydrusen. (**D**) Late-phase ICGA indicating hyperfluorescence corresponding to pachydrusen (black arrowhead). (**E**) Color fundus photograph showing a large pigment epithelial detachment (black arrow), while the pachydrusen remained stable (black arrowhead). (**F**) Swept-source OCT B-scan of the macula showing a large pigment epithelial detachment (white arrow) with subretinal fluid (asterisk) and intraretinal fluid (white arrowhead). (**G**) Color fundus photograph showing the resolution of the pigment epithelial detachment, while the pachydrusen remained stable (black arrowhead). (**H**) Swept-source OCT B-scan of the macula indicating the resolution of the pigment epithelial detachment, subretinal fluid, and intraretinal fluid. FA, fluorescein angiography; ICGA, indocyanine green angiography; MNV, macular neovascularization; OCT, optical coherence tomography.

In exploratory univariable Cox proportional hazards models, polypoidal lesion status was associated with incident MNV (HR, 0.19; 95% CI, 0.04–0.97; likelihood ratio test *p* = 0.029), whereas age, sex, hypertension, smoking, soft drusen, and SFCT were not associated with incident MNV (Table 2). Given the small number of incident MNV events, these results should be interpreted cautiously and considered hypothesis-generating rather than confirmatory. No MNV events occurred among patients with diabetes, resulting in complete separation; therefore, the hazard ratio for diabetes could not be estimated. Two representative cases are shown in Figure 4 and Figure 5.

### 3.2. Five-Year Changes in Pachydrusen Number, Size, and Location

Of 57 eyes with pachydrusen, 19 eyes (mean age, 70.5 years; 15 men and 4 women) completed the 5-year follow-up. We compared the baseline characteristics of eyes that completed the 5-year follow-up with those that did not. Age, sex, hypertension, diabetes, smoking status, and presence of polypoidal lesions did not differ significantly between the 5-year follow-up completers and non-completers (Table 3). Overall, 93 and 101 pachydrusen were detected at baseline and 5 years, respectively. After longitudinal matching of lesions between the two time points, 116 pachydrusen were identified for analysis. Over 5 years, 23 lesions (19.8%) increased in size, 78 (67.2%) remained stable, and 15 (12.9%) decreased in size. There were no lesions that decreased and remained; all decreased lesions disappeared. No RPE atrophy associated with these lesions was observed. At the eye level, 12 eyes were classified as stable (stable group) and 7 as having size changes (changed group, defined as eyes showing either lesion increase in size or decrease in size) (Table 4). In this small subgroup analysis, patients in the changed group showed a higher number of pachydrusen (*p* = 0.02), whereas age, sex, hypertension, diabetes, smoking status, and presence of polypoidal lesions did not differ between the groups.

Based on the concentric ring classification, pachydrusen were predominantly located in the outer zone at baseline (inner circle, 2; intermediate ring, 32; outer zone, 59) and 5 years (inner circle, 1; intermediate ring, 33; outer zone, 67). When dichotomized into locations within the 6000 µm ETDRS grid (inner circle + intermediate ring) and outside the grid (outer zone), 34 (35.6%) and 59 (63.4%) pachydrusen were located within and outside the grid at baseline, respectively. At 5 years, the corresponding numbers were 34 (33.7%) within the grid and 67 (66.3%) outside the grid. The expected proportion outside the grid was defined as the pixel-based ratio of the area outside the grid (213,029 pixels^2^; 36.2%) to the total analyzed area (area within the grid, 376,027 pixels^2^; 63.8%). The observed proportion outside the grid significantly exceeded this expected value at both baseline (63.4%; 95% CI, 53.3–72.5%; exact binomial test, *p* < 0.001) and 5 years (66.3%; 95% CI, 56.7–74.8%; exact binomial test, *p* < 0.001). Using a linear mixed-effects model to evaluate the proportion outside the grid over time, no significant time effect was observed between baseline and 5 years (*p* = 0.27), whereas significant inter-individual variability was noted (*p* = 0.006).

### 3.3. Quadrant-Based Topographic Distribution of Pachydrusen

**Figure 6:**
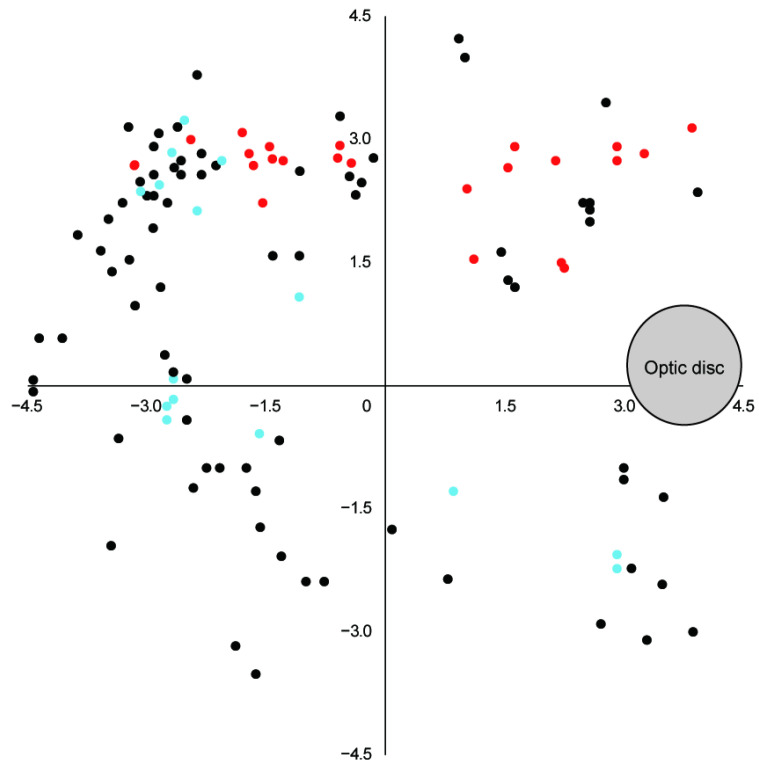
Quadrant-based topographic distribution of pachydrusen plotted on a standardized retinal coordinate map. Each dot represents a pachydrusen lesion (black, stable; red, increased in size; and blue, decreased in size). The gray circle indicates the optic disk.

Quadrant-based topographic analysis was performed using all pachydrusen identified in the baseline and 5-year images (Figure 6). Pachydrusen were most frequently located in the superotemporal quadrant (61/116, 52.6%), followed by the superonasal (22/116, 19.0%), inferotemporal (20/116, 17.2%), and inferonasal (13/116, 11.2%) quadrants. The distribution differed significantly among the four quadrants (*p* < 0.001), with the superotemporal quadrant showing a significantly higher frequency than the other three quadrants combined (*p* < 0.001). In contrast, the quadrant distribution of pachydrusen with size changes did not differ significantly across regions, whether size change was analyzed as a whole (increase or decrease; *p* = 0.18), or separately as increase (*p* = 0.19) or decrease (*p* = 0.76).

## 4. Discussion

In this study, we investigated 5-year longitudinal changes in the morphology and spatial distribution of pachydrusen using diagnostic criteria incorporating late-phase ICGA hyperfluorescence. We found that (1) pachydrusen were significantly more prevalent outside the ETDRS grid, with a predominance in the superotemporal quadrant; (2) eyes with a greater baseline number of pachydrusen were more likely to exhibit size changes over time, and lesion regression was not accompanied by RPE atrophy; and (3) no obvious colocalization between incident MNV and pachydrusen was observed. Collectively, these findings support the possibility that pachydrusen represent a drusen subtype with a pathophysiological basis distinct from that of soft drusen.

Pachydrusen are a drusen subtype associated with the pachychoroid disease spectrum and characterized by choroidal venous dilation and choroidal vascular hyperpermeability (CVH) related to choroidal venous congestion [3,4]. Unlike conventional soft drusen, pachydrusen are predominantly distributed outside the macula [3]. Teo et al. observed that, in fellow eyes with pachydrusen of patients with unilateral nAMD, approximately half of the lesions were located outside a 3000 µm diameter circle centered on the fovea [8]. Nam et al. reported in a hospital-based cohort of pachydrusen eyes without late AMD that 44.5% of lesions were distributed outside a 6000 µm diameter circle centered on the fovea [10]. Sato et al. similarly found in a population-based cohort that approximately half of pachydrusen lesions were located outside a 6000 µm diameter circle [7]. In our study, 63.4% and 66.3% of pachydrusen at baseline and 5 years, respectively, were located outside the 6000 µm diameter circle, significantly exceeding the expected proportion based on the area ratio. This observation is consistent with that of previous studies. Notably, pachydrusen were most frequently located in superotemporal quadrants in this study. Although reports describing quadrant-based asymmetry are limited, this distribution may have been influenced by our diagnostic criteria, which required late-phase ICGA hyperfluorescence. Late-phase ICGA hyperfluorescence, including lesions smaller than 125 µm, has been observed in many eyes with pachychoroid disease, such as central serous chorioretinopathy (CSC) [16] and polypoidal choroidal vasculopathy (PCV) [17], and is frequently detected in central areas of CVH. Several studies have reported vertical asymmetry in the caliber of choroidal veins. Xiao et al. found that choroidal venous dilation is frequently upper-dominant in both CSC and pachychoroid neovasculopathy (PNV) [18]. Conversely, other studies have suggested predominant inferior, particularly inferotemporal, vortex vein engorgement in PCV [19] and the absence of a clear vertical asymmetry in PNV [6]. Given that pachydrusen may be present in pachychoroid eyes before the onset of MNV, they may reflect choroidal hemodynamic characteristics that partially overlap with those observed in CSC. Accordingly, upper-dominant choroidal venous congestion may have contributed to the superotemporal predominance of pachydrusen. Further studies are required to clarify the direct relationship between pachydrusen topography and choroidal venous dilation.

Soft drusen are widely recognized as precursor lesions of nAMD and GA. They comprise extracellular material that accumulates between the RPE and Bruch’s membrane and contain multiple components, including cholesterol [20] and complement pathway proteins [21]. Moreover, variants in genes encoding regulators and complements of the alternative complement pathway have been identified as genetic risk factors for AMD [22,23]. These factors promote chronic inflammation and contribute to the development of MNV and GA. In contrast, the pathophysiology of pachydrusen remains unclear. Although soft drusen regression is frequently accompanied by progression to GA, previous studies have reported that pachydrusen regression is not necessarily accompanied by RPE atrophy [7,10]. Consistent with this finding, none of the 15 regressed lesions in the present study showed RPE atrophy. Given that CVH has been reported to decrease or resolve after the development of venous anastomoses [24], longitudinal fluctuations in pachydrusen size or number may reflect dynamic changes in choroidal hemodynamics. However, the underlying mechanism cannot be determined from the present imaging findings alone, and further investigation is needed. Additionally, we observed no obvious colocalization between the incident MNV and pachydrusen in our study. Previous reports have also shown that the colocalization rate is lower than that observed with soft drusen [8,25], suggesting that pachydrusen may play a more limited role as a direct substrate for MNV development than soft drusen.

Hard drusen exhibit hyperfluorescence on ICGA. Unlike soft drusen, hard drusen are generally not considered precursor lesions of late AMD, and histopathological studies have demonstrated the presence of hyalinized materials [26,27]. Histopathological evaluation of surgically excised PCV lesions also demonstrated hyalinization of choroidal vessels with massive exudation of fibrin and plasma [28]. Hyaline changes are characterized by the deposition and subsequent degeneration of proteinaceous exudates primarily composed of plasma proteins and fibrin. Although no histopathological studies have directly characterized pachydrusen, their association with CVH raises the possibility that hyalinized material derived from choroidal vessels may contribute to the late-phase ICGA hyperfluorescence of pachydrusen. However, not all pachydrusen exhibited late-phase ICGA hyperfluorescence; a previous report demonstrated hyperfluorescence in 69 of 82 eyes (84.1%) [6]. Whether the hypofluorescent lesions belong to the same spectrum or represent a distinct entity remains unclear. Even when lesions appear morphologically similar, their molecular compositions and pathophysiologies may differ, highlighting the need for further investigation.

The present study had some limitations. First, this was a single-center, retrospective, observational study that included only fellow eyes of unilateral nAMD patients, which may have introduced selection bias. In addition, the sample size was limited: only 19 eyes completed the 5-year follow-up, and the number of incident MNV events was small. These factors may have reduced the robustness of the long-term longitudinal assessment and MNV-related analyses. Therefore, the generalizability of our findings should be interpreted cautiously. Second, late-phase ICGA hyperfluorescence was required as part of the diagnostic criteria for pachydrusen. Currently, no standardized diagnostic criteria have been established for pachydrusen, and previous reports have indicated that not all pachydrusen exhibit hyperfluorescence. In addition, the field of view of ICGA is narrower than that of conventional color fundus photography, which may have introduced imaging bias. Accordingly, our criteria may have led to the selection of a subgroup that differed from those defined in earlier studies, which may limit the generalizability of our findings to all pachydrusen. However, incorporating the objective finding of late-phase ICGA hyperfluorescence into the diagnostic definition may have improved the differentiation from soft drusen and enabled a more homogeneous evaluation of pachydrusen over a long-term follow-up. Third, as no histopathological studies on pachydrusen are currently available, the pathological basis of ICGA hyperfluorescence remains unclear. Although we proposed the possibility of hyalinized material, this interpretation is based on indirect evidence and should be considered hypothesis-generating rather than definitive.

## 5. Conclusions

In this 5-year longitudinal study incorporating late-phase ICGA hyperfluorescence into the diagnostic criteria, pachydrusen were predominantly distributed outside the ETDRS grid and showed a superotemporal predilection. Lesion regression was not accompanied by RPE atrophy, and no clear colocalization between pachydrusen and incident MNV was observed. These findings suggest that late-phase ICGA–hyperfluorescent pachydrusen represent a drusen subtype with a pathophysiologic basis distinct from that of soft drusen. Incorporating ICGA-based criteria may provide a homogeneous framework for evaluating the long-term natural history of pachydrusen.

## Figures and Tables

**Table 1:** Clinical characteristics of the study population.

Variable	MNV Event Group (*n* = 8)	Non-Event Group (*n* = 49)
Age (years), mean (SD)	73.6 (9.7)	74.0 (9.1)
Sex (female), No. (%)	3 (37.5)	17 (34.7)
Hypertension, No. (%)	4 (50.0)	24 (49.0)
Diabetes, No. (%)	0 (0.0)	6 (12.2)
Smoking habits (ever-smoker), No. (%)	4 (50.0)	29 (59.2)
Presence of polypoidal lesion, No. (%)	2 (25.0)	31 (63.3)
Presence of soft drusen, No. (%)	2 (25.0)	7 (14.3)
Subfoveal choroidal thickness (μm), mean (SD)	320.3 (139.0)	246.2 (96.0)

MNV, macular neovascularization; SD, standard deviation; No., number.

**Table 2:** Exploratory univariable Cox proportional hazards analysis for incident MNV.

Variable	Univariable HR (95% CI)	*p* (LR Test)
Age (per 1-year increase)	1.01 (0.93–1.10)	0.82
Sex (female vs. male)	1.16 (0.28–4.88)	0.84
Hypertension (yes vs. no)	1.35 (0.33–5.41)	0.68
Diabetes (yes vs. no)	Not estimable *	—
Smoking (ever-smoker vs. never)	0.78 (0.19–3.11)	0.72
Polypoidal lesion (present vs. absent)	0.19 (0.04–0.97)	0.029
Soft drusen (present vs. absent)	1.21 (0.24–6.04)	0.82
Subfoveal choroidal thickness (per 10 µm increase)	1.05 (0.98–1.11)	0.15

* Not estimated because no events occurred in the diabetes group (complete separation). CI, confidence interval; HR, hazard ratio; LR, likelihood ratio.

**Table 3:** Baseline characteristics of the 5-year follow-up completers and non-completers.

Variable	5-Year Completers (*n* = 19)	Non-Completers (*n* = 38)	*p*
Age (years), mean (SD)	70.5 (6.2)	73.9 (9.6)	0.06
Sex (female), No. (%)	4 (21.1)	16 (42.1)	0.15
Hypertension, No. (%)	8 (42.1)	20 (52.6)	0.58
Diabetes, No. (%)	4 (21.1)	2 (5.3)	0.09
Smoking habits (ever-smoker), No. (%)	13 (68.4)	20 (52.6)	0.39
Presence of polypoidal lesion, No. (%)	13 (68.4)	20 (52.6)	0.39

SD, standard deviation; No., number.

**Table 4:** Baseline characteristics of the stable and changed groups.

Variable	Stable Group (*n* = 12)	Changed Group (*n* = 7)	*p*
Age (years), mean (SD)	71.6 (6.6)	68.7 (5.3)	0.47
Sex (female), No. (%)	3 (25.0)	1 (14.3)	1.00
Hypertension, No. (%)	6 (50.0)	2 (28.6)	0.63
Diabetes, No. (%)	3 (25.0)	1 (14.3)	1.00
Smoking habits (ever-smoker), No. (%)	8 (66.7)	5 (71.4)	1.00
Presence of polypoidal lesion, No. (%)	9 (75.0)	4 (57.1)	0.62
Number of pachydrusen, mean (SD)	3.3 (2.4)	7.7 (5.5)	0.02

SD, standard deviation; No., number.

## Data Availability

The data used to support the findings of this study are restricted by the Kawasaki Medical School Ethics Committee to protect patient privacy. Data are available from Hiroyuki Kamao [hironeri@med.kawasaki-m.ac.jp] for researchers who meet the criteria for access to confidential data.
